# Comparative Transcriptomic and Physiological Analyses Reveal Key Factors for Interstocks to Improve Grafted Seedling Growth in Tangor

**DOI:** 10.3390/ijms24076533

**Published:** 2023-03-31

**Authors:** Yi Rong, Ling Liao, Sichen Li, Wen Wei, Xiaoyi Bi, Guochao Sun, Siya He, Zhihui Wang

**Affiliations:** 1 College of Horticulture, Sichuan Agricultural University, Chengdu 611130, China; 2 Citrus Research Institute, Southwest University, Chongqing 400715, China; 3 Institute of Pomology and Olericulture, Sichuan Agricultural University, Chengdu 611130, China

**Keywords:** grafting, interstock, rootstock, tangor, Mingrijian, transcriptome

## Abstract

Interstock is an important agronomic technique for regulating plant growth and fruit quality, and overcoming the incompatibility between rootstocks and scions; however, the underlying mechanisms remain largely unknown. In this study, the effects and regulatory mechanisms of tangor grafting, with and without interstocks, on the growth and development of scions were analyzed by combining morphology, physiology, anatomy and transcriptomics. Morphological and physiological analyses showed that interstocks (‘Aiyuan 38’ and ‘Daya’) significantly improved the growth of seedlings, effectively enhanced the foliar accumulation of chlorophyll and carotenoids, and increased the thickness of leaf tissues. Using ‘Aiyuan 38’ as the interstock, photosynthetic efficiency and starch content of citrus seedlings improved. Transcriptomics showed that genes related to photosynthesis and photosynthetic antenna proteins were upregulated in interstock-treated seedlings, with significant upregulation of photosystem PSI- and PSII-related genes. In addition, multiple key genes may be involved in plant hormone signaling, starch and sucrose metabolism, and transcriptional regulation. Taken together, these findings provide novel insights into the role of interstocks in regulating and contributing to the growth and development of grafted seedlings, and will further define and deploy candidate genes to explore the mechanisms of rootstock-interstock-scion interactions.

## 1. Introduction

Top grafting is the most effective way to renew citrus varieties and rapidly adapt them for market adjustments. Interstock is an inevitable common agronomic measure during top grafting. During production, part of the scion of the original adult tree is often used as an interstock, and new varieties are replaced at higher branches to form a new scion for fruit production. The interstock is a segment of a tree trunk grafted between the rootstock and scion [1,2]. Interstock has been applied in persimmons [3], lemons [2], peaches [4], apples [5], and other fruit trees to regulate tree size [4], improve fruit yield and quality, and delay tree aging [2]. Rational use of a combination of citrus rootstocks and interstocks can enhance disease resistance [6,7], regulate production period [8] and tree dwarfing [5], and improve citrus fruit quality [9,10]. Initially, interstocks were studied as tools to overcome the interspecific incompatibility between rootstocks and scions [11]. For instance, ‘Hongmian miyou’ was incompatible with trifoliate rootstocks, such as *Poncirus trifoliate*; thus, an interestock of ‘Guanxi miyou’ (compatible with *Poncirus trifoliata*) was commonly used between the scion (‘Hongmian miyou’) and rootstock (*Poncirus trifoliata*) [12]. Interstocks regulate the growth and fruiting of scions, and overcome the incompatibility between the rootstock and scion by altering endogenous hormones [12,13], mineral elements [14], secondary metabolites [2], enzyme activity [15] and related gene expression patterns [16].

Citrus is one of the most important fruit crops worldwide, and it is widely cultivated in the tropical, subtropical and Mediterranean regions [17]. In recent decades, the scale of citrus cultivation has expanded rapidly, particularly of the tangor type (*Citrus reticulata* × *Citrus sinensis*, mandarin-orange hybrid), which combines the benefits of orange and mandarin, and has become increasingly popular among consumers [18]. ‘Aiyuan 38’ (*Citrus reticulata*, A) and ‘Daya’ (*Citrus sinensis* (L.) Osbeck × *Citrus reticulata* Blanco cv. ‘Ponkan’, D), a high-quality tangor, has been widely cultivated in the Sichuan Province over the past decade. With the development of the citrus breeding industry, an increasing number of high-quality mandarin-orange hybrid varieties have been produced. For instance, ‘Mingrijian’ (M), also known as ‘Asumi’ in Japan, originated from Japan and was introduced to China in 2011, which is a mandarin hybrid obtained by crossing ‘Harumi’ (*Citrus reticulata* × (*Citrus reticulata* × *Citrus sinensis*)) and ‘Sweet spring tangelo’ (*Citrus reticulata* cv. Unshiu × *Citrus Hassaku* Hort. ex Tanaka). Moreover, ‘Hongju’ (*Citrus reticulata* Blanco cv. Rad tangerine, H), which is commonly used as a rootstock, promotes grafting affinity or grafting compatibility, and has been widely used in the production of medium-ripe citrus varieties in most regions of China [19].

In recent years, rootstock research in the citrus context has become an important topic [20]. However, little information is available regarding the direct effects of interstocks on scion physiology and the mechanisms of rootstock–interstock–scion interaction. In this study, morphological, physiological, anatomical and transcriptomic analyses were performed on seedlings with interstock grafting (HAM, ‘Hongju’/‘Aiyuan 38’/‘Mingrijian’, and HDM, ‘Hongju’/‘Daya’/‘Mingrijian’,) and seedlings without interstock grafting (HM, ‘Hongju’/‘Mingrijian’) to explore the key mechanisms underlying the interstock-mediated regulation of citrus growth and development. We hypothesized that when ‘Hongju’ was used as the base rootstock, using ‘Aiyuan 38’ or ‘Daya’ as an interstock would improve the growth of ‘Mingrijian’ tangor. Thus, our objectives were to exploit the material basis and gene expression patterns, and illustrate the biometabolic pathways playing regulatory roles in tangors with and without interstock grafting during seedling growth. The large-scale, multi-strategy datasets produced during this work are expected to offer valuable resources for understanding the mechanism of interstocks in citrus and the application of interstocks in horticultural plants.

## 2. Results

### 2.1. Morphological Changes

**Figure 1:**
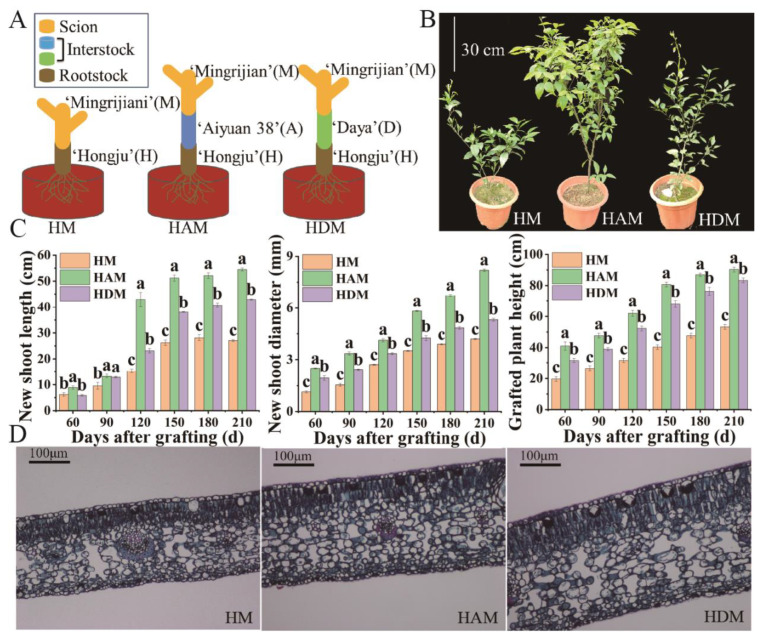
The effect of interstocks on the growth responses of ‘Mingrijian’ citrus. (**A**) Grafting diagram of seedlings used in this experiment. (**B**) Effects of interstocks on the growth morphology of ‘Mingrijian’ after 210 days. (**C**) The length of new shoots, diameter of new shoots, and plant height of grafted seedlings under different interstocks. Abscissa coordinates represent different days after grafting. Longitudinal coordinates represent the increment of indexes. Data were the mean ± standard error of three replicates. Error bars indicate the standard error of total growth, and different letters indicate significant differences in total growth, *p* < 0.05. (**D**) Leaf cross-cutting structure (×200) of ‘Mingrijian’ after 210 days of grafting onto different interstocks.

From 60 to 210 days after grafting, significant differences in plant growth and phenotype were observed among the different treatments. Compared with HM, HAM and HDM bore more branches and leaves; meanwhile, HAM bore more lush branches and leaves than HDM (Figure 1B). The shoot length, shoot diameter and plant height of HAM were the highest, whereas those of HM were the lowest among all treatments (Figure 1C). The growth of interstock-treated plants was significantly improved compared to that of control plants, suggesting that interstock played a key role in the growth of ‘Mingrijian’ seedlings. To understand the microstructural differences between grafted combinations, leaf sections of the grafted plants were observed under a light microscope (Figure 1D). The leaf anatomical structures of the three grafted combinations were similar; however, leaf tissues, including the lower leaf epidermis, palisade tissue and spongy tissue, were significantly thicker in HAM and HDM than in HM (Table S1). Leaf thickness in HAM and HDM was 24.7% and 46.0% greater than that in HM, respectively.

### 2.2. Changes in Photosynthetic Indexes and the Related Physiological Indexes

**Figure 2:**
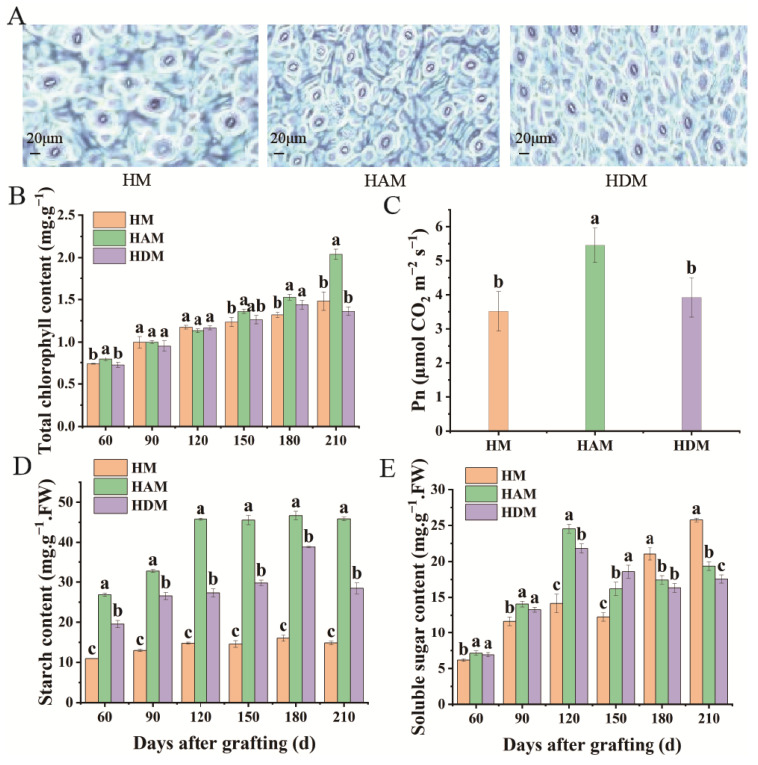
Effect of different interstocks on photosynthetic related physiological indicators of grafted ‘Mingrijian’ seedlings leaves. (**A**) Microscopy of stoma from plants after 210 days, (**B**) total chlorophyll, (**C**) Pn, (**D**) starch content and (**E**) soluble sugar content. Data were the mean ± standard error of three replicates. Error bars indicate the standard error, and different letters indicate significant differences, *p* < 0.05.

The abaxial surfaces of leaf samples were scanned (Figure 2A). Leaf stomatal density, width, aperture, and area in HAM and HDM were higher than those in HM; however, no significant differences in stomatal length were observed among the three treatments. Except for stomatal length, stomatal density, width, aperture and area in HAM were higher than those in HDM (Figure S1). Thus, interstock altered the stomatal parameters and improved the stomatal status of leaves. When ‘Aiyuan 38’ (‘A’) was used as an interstock, the best improvement effect on leaf stomata was noted. Chlorophyll a, chlorophyll b, total chlorophyll and carotenoid content in HAM were the highest among the three combinations, whereas chlorophyll a/b ratio was the lowest (Figure 2B and Figure S2), indicating that the plants grafted with ‘A’ as an interstock exhibited better photosynthetic capacity and stronger shade tolerance. Photosynthetic pigment analysis showed that interstock affected the accumulation of photosynthetic pigments in the scion to a certain extent. The net photosynthetic rate (Pn), transpiration rate (Tr), and stomatal conductance (Gs) were higher, but the intercellular CO_2_ concentration (Ci) was lower in HAM than in HM. However, no significant differences were noted in Pn and Gs between HDM and HM (Figure 2C and Figure S3). Simultaneously, Pn and Tr in HAM were higher than those in HDM. However, Ci and Gs levels did not differ significantly between HAM and HDM. Therefore, interstock enhanced the photosynthetic capacity of scions to a certain extent, although the plants grafted with ‘A’ as an interstock showed better photosynthetic capacity. To further determine the effect of interstock on photosynthetic capacity, we examined the starch and soluble sugar contents of seedlings. Starch content of HAM and HDM was significantly higher than that of HM (Figure 2D). Thus, interstock enhanced the accumulation of photosynthetic products in leaves. In addition, the soluble sugar content of HAM and HDM increased initially and decreased subsequently (Figure 2E). Compared with late-stage HM seedlings, interstock significantly decreased soluble sugar levels in leaves, indicating that more soluble sugars were used for vigorous seedling growth.

### 2.3. Changes in Endogenous Hormones

**Figure 3:**
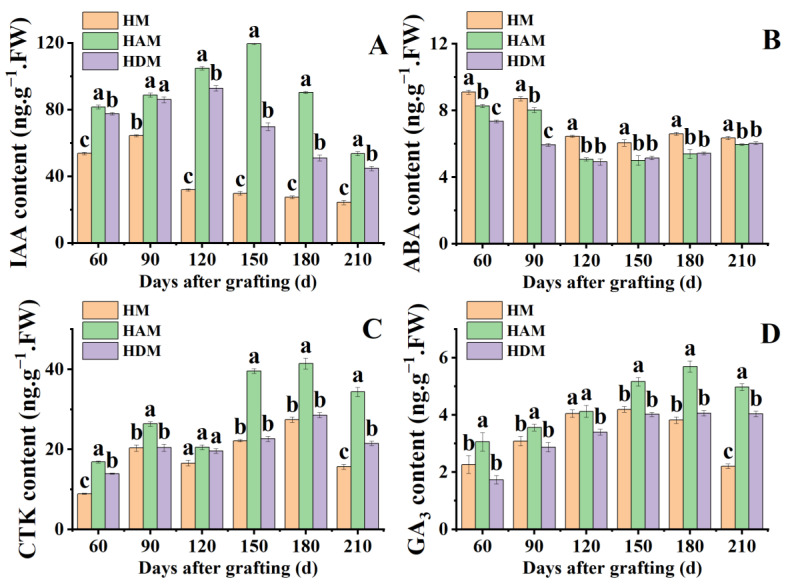
The phytohormone levels in leaves under different interstock treatments. (**A**) IAA: indole-3-acetic acid, (**B**) ABA: abscisic acid, (**C**) CTK: cytokinin, (**D**) GA_3_: gibberellin A_3_. Data were the mean ± standard error of three replicates. Error bars indicate the standard error, and different letters indicate significant differences, *p* < 0.05.

To explore the effect of interstocks on plant hormones, we analyzed the changes in ABA, IAA, GA_3_ and CTK content. Interstock significantly increased IAA, GA_3_ and CTK accumulation (Figure 3). Except IAA at 90 days and CTK at 120 days after grafting, IAA, GA_3_ and CTK levels in HAM were significantly higher than those in HDM. In HAM, IAA, GA_3_ and CTK levels peaked at 120, 150 and 180 days after grafting, respectively, followed by a decrease. However, GA_3_ levels changed little after reaching a peak at 180 days after grafting in HDM. At 210 days after grafting, IAA level accumulation in HAM and HDM increased by 98.4% and 83.6%, respectively, compared to those in HM. Notably, leaf CTK levels in HAM were significantly higher than those in HM, except at 120 days after grafting. Compared with HM, interstock significantly reduced ABA accumulation. However, there were no significant differences in ABA levels between HAM and HDM from 120 to 210 days after grafting. Overall, interstock altered the accumulation of phytohormones and significantly enhanced the accumulation of hormones related to the promotion of plant growth and development, such as IAA, GA_3_ and CTK. However, different interstocks produced varying effects on hormone accumulation. Among these, ‘A’ as an interstock showed the most significant promoting effect.

### 2.4. Transcriptome Analysis and Enrichment Analysis

The Illumina platform was used to construct nine cDNA libraries, and the average clean data obtained for each sample were more than 6 GB (Q20 > 96% and Q30 > 91%) (Table S2). Cluster heat maps of three biological replicates for all treatments were presented in Figure S4. Clean data were deposited in the NCBI SRA database. The mapping rate of all nine sequencing samples was between 89.4% and 92.5% (Table S3). Overall, RNA-seq data produced were of excellent quality and were used for further research.

**Figure 4:**
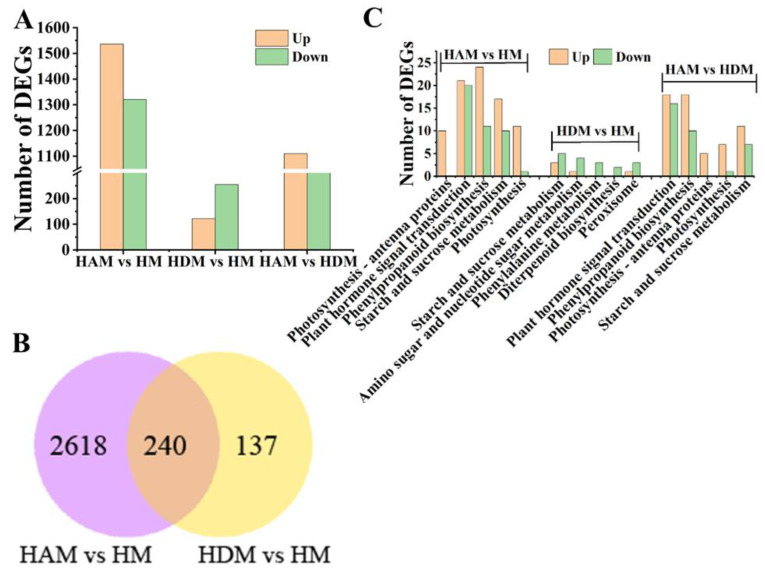
Overview of transcriptomics changes of different comparisons affected by interstocks. (**A**) DEGs in three comparisons. (**B**) Venn diagram of DEG numbers in different comparison groups. The number on the left of the red arrow indicates upregulated gene number, and the number on the left of the green arrow indicates downregulated gene number. (**C**) KEGG analysis (top 5) in three comparisons.

We noted 1537 upregulated and 1321 downregulated differentially expressed genes (DEGs) in the HAM vs. HM comparison, and 122 upregulated and 255 downregulated DEGs in the HDM vs. HM comparison (Figure 4A and Table S4). Therefore, interstock altered scion transcriptome profiles. In addition, 2063 DEGs were identified in the HAM vs. HDM comparison, suggesting that ‘A’ as an interstock produced a stronger effect on the transcriptome of scions. Further, DEGs that overlapped among different comparisons were analyzed. A total of 240 overlapping DEGs were noted between HAM vs. HM, and HDM vs. HM (Figure 4B), which may be related to the vigorous growth of grafted seedlings caused by interstock. The 12 DEGs were analyzed randomly using qRT-PCR to validate the reliability of the RNA-seq data, and high accuracy and repeatability of the RNA-seq results were confirmed (Figure S5).

To highlight their potential biological functions, the identified DEGs were subjected to gene ontology (GO) enrichment analysis. GO enrichment analysis showed that DEGs from the HAM vs. HM, HDM vs. HM, and HAM vs. HDM comparisons were assigned 660, 315, and 602 GO terms, respectively (Table S5). In the cellular component category, “membrane” (photosynthetic membrane, thylakoid, thylakoid part, extrinsic component of membrane and thylakoid membrane) and “photosystem” were the most abundant terms in the HAM vs. HM, and HAM vs. HDM comparisons. Moreover, in the molecular function category, “enzyme inhibitor activity”, “enzyme regulator activity” and “molecular function regulator” were the most abundant terms in the HAM vs. HM, HDM vs. HM, and HAM vs. HDM comparisons.

KEGG pathway analysis was used to further reveal the potential functions of DEGs (Table S6). The identified DEGs in the HAM vs. HM, and HAM vs. HDM comparisons were significantly enriched in photosynthesis–antenna proteins, photosynthesis, plant hormone signal transduction and phenylpropanoid biosynthesis (Figure 4C). Moreover, DEGs involved in phenylalanine, starch and sucrose metabolism were identified in the HAM vs. HM and HDM vs. HM comparisons. The results of annotation showed that highly representative pathways and GO terms, such as photosynthesis, photosynthesis–antenna proteins and hormone signal transduction, may be key factors in the interstock-mediated regulation of scion development.

### 2.5. Transcription Factors

**Figure 5:**
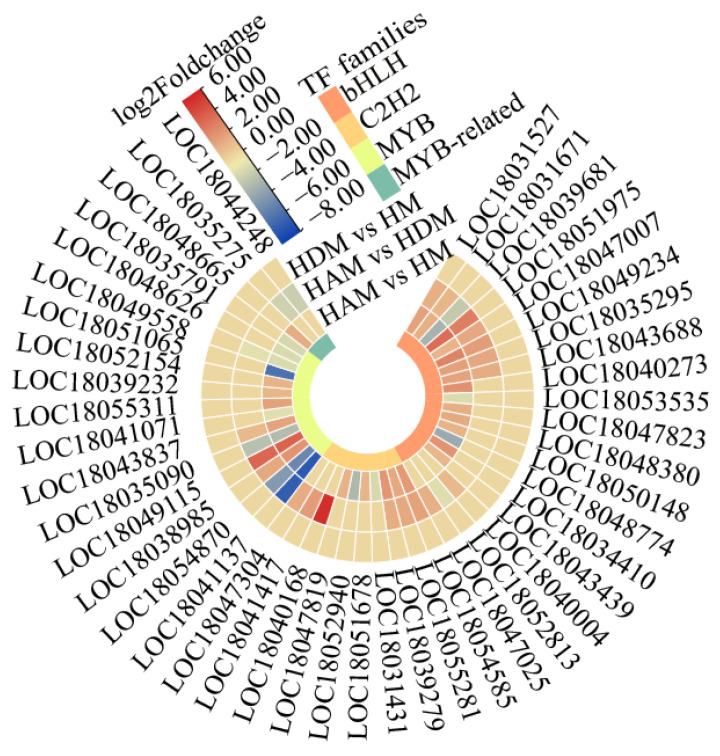
Heat map shows the differentially expressed genes in the bHLHs, C2H2s and MYBs transcription factor families. Heat map of DEGs was drawn using the log2 fold-change value obtained from the pairwise comparison of samples. Red and blue indicate upregulation and downregulation, respectively.

We identified 150 and 97 differentially expressed transcription factors in the HAM vs. HM, and HAM vs. HDM comparisons, respectively (Table S7). Most genes encoding transcription factors were upregulated. We investigated main plant transcription factor families (Figure 5). Overall, bHLH, HB, MYB and C2H2 transcription factors were strongly represented in the HAM vs. HM comparison (Figure S6). The bHLH family had the most DEGs, followed by the HB and MYB families, with 14 DEGs each. In the HAM vs. HDM, of the 97 differentially expressed transcription factors, the bHLH family accounted for the highest proportion (Table S7). Most genes encoding the bHLH and C2H2 transcription factors were upregulated in the HAM vs. HM, and HAM vs. HDM comparisons. More MYB transcription factor genes were upregulated in HAM vs. HM than in HAM vs. HDM. Therefore, the expression of many transcription factor genes was affected by interstocks, implying that interstocks played a key role in scion growth.

### 2.6. Genes Involved in Photosynthesis–Antenna Proteins and Photosynthesis

**Figure 6:**
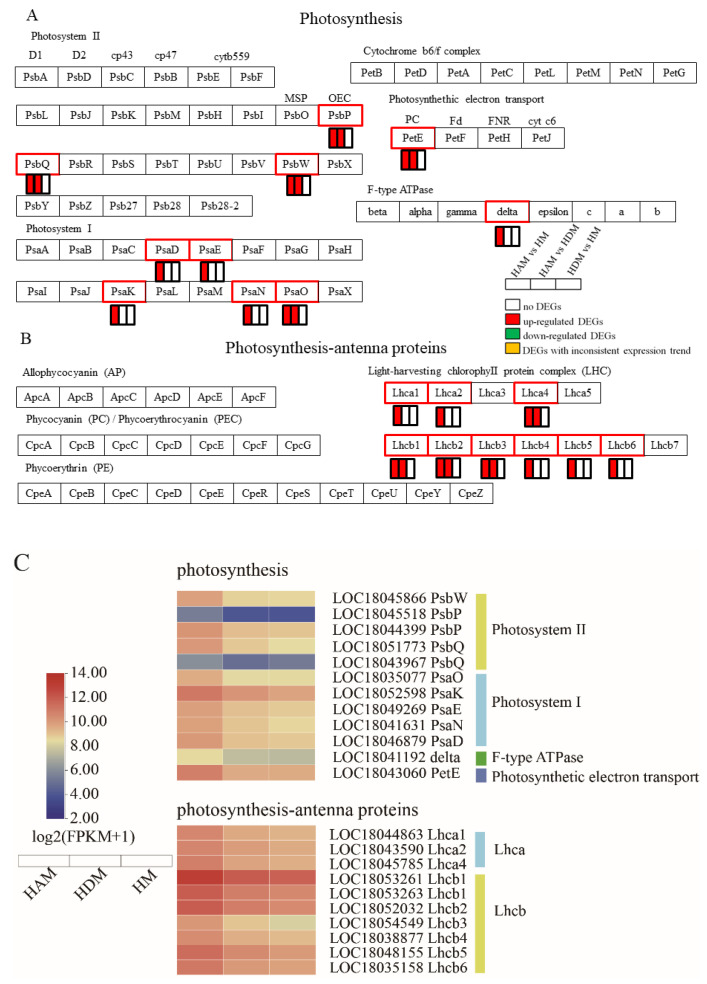
Interstocks induced changes in the expression profiles of photosynthesis-related genes in three comparisons. (**A**) Photosynthesis pathway genes, (**B**) photosynthesis–antenna proteins pathway genes, and (**C**) heat map of photosynthesis-related gene expression. The rectangles behind the gene, which were tagged with red, green, white and yellow color, represent the upregulated DEGs, downregulated DEGs, unchanged DEGs, and the DEGs with inconsistent expression trends, respectively.

Interstocks promoted the expression of most photosystem (PS)-related genes in the photosynthetic pathway (Table S8 and Figure 6A). Specifically, in PSI, PsaD, PsaE, PsaK, PsaN and PsaO were upregulated in the HAM vs. HM; meanwhile, in HAM vs. HDM, only PsaO was upregulated, but their expression of the other genes remained unchanged. In PSII, most transcripts of PsbP, PsbQ and PsbW were upregulated in the HAM vs. HM, and HAM vs. HDM, although the degree of upregulation was greater in the former comparison. One photosynthetic electron transporter (PetE) was upregulated in the HAM vs. HM, and HAM vs. HDM comparisons, but not in the HDM vs. HM comparison, and its transcript abundance was higher in the HAM vs. HM than in the HAM vs. HDM. Moreover, only one gene encoding an F-type ATPase (delta) was upregulated in the HAM vs. HM, whereas its expression remained unchanged in the HAM vs. HDM, and HDM vs. HM.

Furthermore, in terms of photosynthesis–antenna proteins, interstocks significantly enhanced the photosynthesis potential of the scion, greatly improving the “energy source” for plant morphogenesis (Table S8 and Figure 6B). Ten genes (Lhca1, Lhca2, Lhca4, 2 Lhcb1, Lhcb2, Lhcb3, Lhcb4, Lhcb5 and Lhcb6) were upregulated in the HAM vs. HM; meanwhile, five genes (Lhca4, 2 Lhcb1, Lhcb2 and Lhcb3) were upregulated in the HAM vs. HDM, although their expression remained unchanged in the HDM vs. HM. Therefore, these results indicated that interstock enhanced the photosynthetic capability of scions. Moreover, compared with ‘D’, ‘A’ as the interstock effectively improved the photosynthesis of the scion.

### 2.7. Genes Involved in Phytohormone Signaling

**Figure 7:**
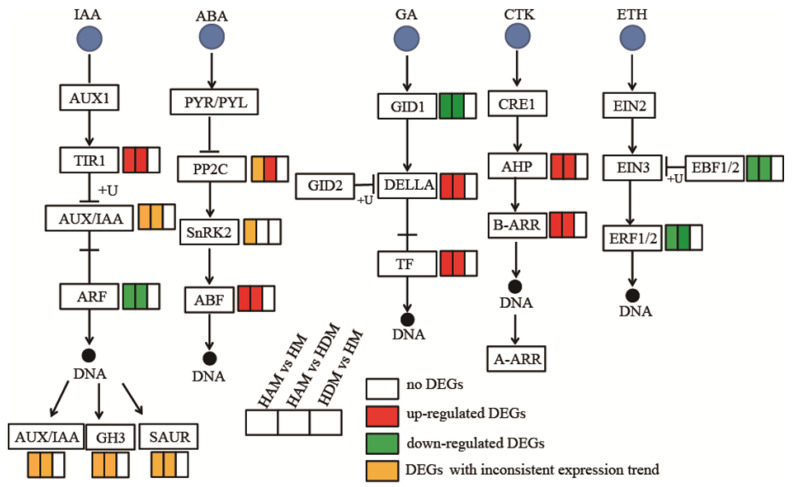
Interstocks induced changes in the expression profiles of hormone signaling pathway genes in three comparisons. The rectangles behind the gene, which were tagged with red, green, white and yellow color, represent the upregulated DEGs, downregulated DEGs, unchanged DEGs and the DEGs with inconsistent expression trends, respectively.

To explore the possible molecular mechanism underlying interstock-mediated regulation of scion growth, we investigated the expression profiles of genes involved in plant hormone signaling (Table S9 and Figure 7).

Regarding IAA signal 15 and 13 DEGs related to auxin signaling were detected in the HAM vs. HM, and HAM vs. HDM, respectively. In both comparisons, only one negative regulator, AUX/IAA (IAA1 in HAM vs. HM, and IAA14 in HAM vs. HDM), was upregulated, whereas the other AUX/IAAs were downregulated. More AUX/IAAs were downregulated than upregulated in both comparisons. Meanwhile, AUX/IAA expression remained unchanged in HDM vs. HM. In both HAM vs. HM and HAM vs. HDM, TIR1 (LOC18052162) was upregulated, one GH3 gene (LOC18053209) was downregulated, and three other GH3 genes (LOC18038130, LOC18035901, and LOC18054772) were upregulated. SAURs (small auxin-up RNA) were both upregulated and downregulated, and the number of up/downregulated genes differed between the HAM vs. HM, and HAM vs. HDM.

Regarding ABA signal, only one negative regulator, PP2C (LOC18034994), was downregulated in HAM vs. HM, whereas other PP2Cs were upregulated in HAM vs. HDM, and HAM vs. HM. Additionally, ABF gene expression was induced in both HAM vs. HDM, and HDM vs. HM. Notably, the positive regulators, SnRK2, were upregulated (LOC18037095) and downregulated (LOC18049391) in HAM vs. HM, although their expression remained unchanged in HAM vs. HDM, and HDM vs. HM.

Regarding GA signal, genes encoding DELLA proteins (GAI) and TF were upregulated, whereas the GA-insensitive DWARF1 GID1 (GA receptor) was downregulated in both HAM vs. HM, and HAM vs. HDM comparisons.

Regarding CTK signal, a histidine-containing phosphotransfer protein (AHP) and the downstream transcription activator A/B-Arabidopsis response regulator (ARR) were significantly upregulated in the HAM vs. HM, and HAM vs. HDM comparisons. Interestingly, no DEGs involved in CTK signaling were detected in the HDM vs. HM comparison.

ERF1B and EBF1, which are hub genes involved in ethylene signaling regulation, were downregulated in both HAM vs. HM, and HAM vs. HDM comparisons, although their expression remained unchanged in the HDM vs. HM comparison.

Genes involved in other phytohormones showed marked changes under the influence of interstock (Figure S7). TIFY 10A (LOC18036545), involved in JA signaling, and NPR1 (LOC18031816) and PR1 (LOC18033982), involved in SA signaling, were downregulated in the HAM vs. HM comparison. Moreover, genes involved in the BR pathway were upregulated in the HAM vs. HM, and HAM vs. HDM comparisons.

### 2.8. Genes Involved in Starch and Sucrose Metabolism, Amino Sugar and Nucleotide Sugar Metabolism, and Other Glycan Degradation Pathways

**Figure 8:**
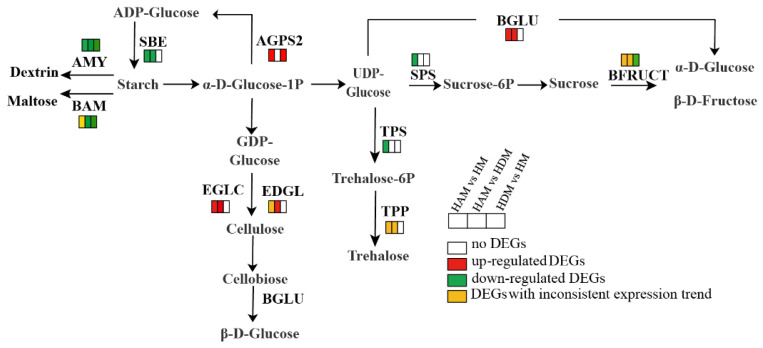
Interstocks induced changes in the expression profiles of the key starch and sugar metabolism. The rectangles behind the gene, which were tagged with red, green, white and yellow color, represent the upregulated DEGs, downregulated DEGs, unchanged DEGs and the DEGs with inconsistent expression trends, respectively.

We dissected the profiles of genes involved in starch and sucrose metabolism, amino sugar and nucleotide sugar metabolism, and other glycan degradation pathways (Figure 8 and Table S10). In starch and sucrose metabolism pathways, 27, 18 and 8 genes were differentially expressed between the HAM vs. HM, HAM vs. HDM, and HDM vs. HM groups, respectively. Specifically, three genes encoding endoglucanase (EDGL), four genes encoding beta-glucosidase (BGLU) and four genes encoding endoglucan-1, 3-beta-glucosidase (EGLC) were upregulated in HAM vs. HM (Table S10). Meanwhile, three genes encoding endoglucanase (EDGL), three genes encoding β-glucosidase (BGLU) and three genes encoding glucan-endonuclosidase (EGLC) were upregulated in HAM vs. HDM. However, the expression of these genes remained unchanged in HDM vs. HM. Only one gene encoding sucrose phosphate synthase (SPS1, LOC18054473) was downregulated in HAM vs. HM, but no related genes were found in the other two groups. The α-amylase (AMY) was downregulated in all three comparisons. Only one β-amylase (BAM1, LOC18032167) gene was upregulated in HAM vs. HM, while the other BAM genes were downregulated in all three comparisons. In addition, the transcriptional profiles of genes involved in amino and nucleotide sugar metabolism were investigated. Most genes were upregulated in HAM vs. HM. However, fewer genes were involved in this metabolic pathway in HDM vs. HM, and HAM vs. HDM. Only one gene encoding chitinase was downregulated in HAM and HDM. In other glycan degradation pathways, most genes in HAM vs. HM, and HAM vs. HDM were upregulated. However, the expression of these genes remained unchanged in HDM vs. HM.

## 3. Discussion

Grafting is a common technique in citriculture. Rootstocks control plant development, improve fruit quality, yield and plant nutrient uptake, and enhance tolerance or resistance of abiotic and biotic stress [21,22,23]. The scion and rootstock combination is an efficient method enhancing the productivity and disease resistance of a certain cultivar [24]. Rootstocks and interstocks affect citrus fruit quality at maturity [2]. However, knowledge of the effects of interstocks on citrus scions during seedling development is limited, particularly at the molecular level. In this study, we compared physiological and transcriptome changes resulting from two distinct scion–interstock combinations to a non-interstock-grafted control. Our findings demonstrated that interstocks induced significant changes in citrus scions.

Seasonal variation in plant growth rate varied, with the most noticeable difference noted during rapid shoot growth. Beginning in mid-July, there were clear disparities in scion growth among the three grafted groups. This was one of the stages when interstocks produced a substantial impact on growth. Based on the above findings, interstocks were in a condition of accelerated cell proliferation and metabolism. Generally, carbon allocation can affect plant growth and development. Here, HAM and HDM gathered more starch, but showed lower soluble sugar levels than HM. Additionally, HAM scions stored higher starch and less soluble sugar than HDM scions, implying that ‘A’ had an independent effect on carbohydrate allocation. Furthermore, the lower the growth rate of grafted seedlings, the greater the soluble sugar accumulation in leaves; the higher the growth rate, the greater the soluble sugar consumption. Many studies have demonstrated the importance of sugars as signaling molecules able to recognize nutritional status and regulate the processes of development and growth properly [25]. Sugar, for instance, increased lateral meristem elongation in rose [26] and sorghum [27]. Further, low glucose and fructose levels in plants with ‘M9’ interstock were shown to significantly affect the physiology of both rootstock and scion [28]. Based on our observations, interstocks likely served critical regulatory roles in recognizing and regulating the appropriate ratio of starch and soluble sugar for metabolic activities, ensuring scion growth at the expense of excess starch reserves.

In this study, 2063, 2858 and 377 DEGs were identified in HAM vs. HDM, HAM vs. HM and HDM vs. HM, respectively. A small number of DEGs were found in the HDM vs. HM combination, potentially attributed to the extremely comparable genetic backgrounds of ‘Daya’ and ‘Mingrijian’. In previous studies, a limited number of DEGs were reported in other grafting combinations [29,30], similar to our results. Interstock-induced changes in shoot structure appeared to be controlled by a few genes, as evidenced by interstock-induced dwarfism in apple and sweet persimmon [3,13]. Our results showed that interstocks affect the growth of scions by regulating gene expression, even if only a few genes were involved. GO and KEGG analysis indicated that the detected unigenes were engaged in various pathways, such as plant hormone signaling and photosynthetic metabolism. The potential genes responsible for the interstock influence on scion growth were probably involved in the photosynthesis and GA, IAA and CTK signaling.

Photosynthesis was recognized as an exceedingly sophisticated and crucial system, as it must balance the light energy absorbed by the photosystems with the energy spent by the plant growth metabolism [31]. It was obvious that the rates of source and sink activities must be balanced for optimal plant growth and development.

Chlorophyll was essential for photosynthesis as it formed the main part of pigment-protein complexes [32]. Chlorophyll a, b and a + b levels were not significantly different between HDM and HM in the research. Nevertheless, chlorophyll a, b and a + b levels were substantially higher in HAM than in HM. Sun plants exhibited a higher chlorophyll a/b ratio [33], but an opposite finding was noted in the research. HAM had a higher photosynthetic rate and lower chlorophyll a/b ratio than HM. Although the difference in chlorophyll a/b ratio between HDM and HM was not significantly different, the value in HDM was somewhat lower than that in HM. Therefore, interstocks may have improved the shade tolerance of ‘Mingrijian’ leaves, similar to the results reported previously [34]. Carotenoids served as crucial photosynthetic pigments [35] owing to their roles in light gathering, energy transfer, quenching and photoprotection [36]. As a result, a decline in carotenoid content will directly suppress photosynthesis [37]. The carotenoid levels were greater in HAM than in HM in this study. Moreover, the photosynthetic parameters (except Ci) of interstock-grafted ‘Mingrijian’ were enhanced. Overall, interstock could strengthen scion photosynthetic capacity. Similarly, in a previous study, grafting enhanced the photosynthetic capacity in citrus [38].

The initial phase in photosynthesis is light harvesting [39]. Antenna proteins, found in LHC protein complexes, serve as a peripheral antenna system, efficiently gathering light and providing photoprotection [40]. LHC gene expression was raised in response to intense light, implying that these genes had a photoprotective function [41]. In the present study, 10 and 5 Lhc genes, which encoded chlorophyll a-b binding proteins, were upregulated in HAM vs. HM and HAM vs. HDM, respectively, suggesting that interstock may play a key role in photoprotection and light capture by improving the light-harvesting potential of ‘Mingrijian’ leaves. Most photosynthetic pigments in vascular plants are controlled by LHC proteins [42]. Quick modification of leaf photosynthetic machinery and capacity can take place in response to changes in light availability [43]. Four multi-subunit protein complexes play a major role in light reactions [44]. In transcriptome analysis, transcript abundance of some genes in the photosynthetic pathway differed, which may alter the photosynthetic indices of ‘Mingrijian’ after interstock-grafting. Psa and Psb are core proteins crucial for the functional assembly of PSI and PSII [45]. Upregulation of Psa maintains PSI stability and avoids the reduction in the photosynthetic rate. In this study, the five upregulated Psas and five upregulated Psbs produced by interstocks enhanced chlorophyll binding. Overall, these results indicated that interstock strengthened photosynthetic electron transport and stability of PSI and PS II.

Crosstalk among phytohormone signaling pathways creates a complex network of overlapping signaling [46], ultimately regulating plant growth and development [47]. In our present study, plant hormone levels and hormone-related gene expression were significantly altered in scion in the presence of interstocks (Figure 3 and Figure 7). Compared with ‘Daya’ as the interstock, ‘Aiyuan 38’ produced more pronounced alterations. Therefore, interstocks produced varying effects on transcript expression and hormone levels in grafted plants.

Auxin is essential for plant growth and development [48]. The key classes of principal auxin response genes are AUX/IAA, GH3 and SAUR [49]. In our study, interstocks markedly decreased Aux/IAA gene expression, while increasing the expression of the auxin receptor TIR1. In addition, GH3, which facilitated auxin breakdown, was highly expressed. GH3 expression was induced by auxin and controlled by a negative feedback loop. This gene most likely facilitates the maintenance of auxin balance, but does not prevent other aspects of plant growth. In fact, a higher IAA concentration was reported in interstock-grafted plants than in non-interstock-grafted ones. Therefore, interstock may regulate plant growth by controlling the interplay of Aux/IAA, GH3, and TIR1, consistent with previous reports [50]. As such, Aux/IAA multimers degradation would efficiently boost auxin signaling, and higher auxin levels may strengthen plant growth.

In GA signaling, DELLA proteins were the major suppressors that bind the essential downstream regulators to hinder GA signal transduction [51]. DELLA proteins are ubiquitinated and degraded as a response to GA [52]. In this study, GA signal transduction was more active in HAM vs. HM. Specifically, PIF4 expression was significantly uregulated, whereas DELLA protein gene expression was unchanged. Furthermore, we analyzed the GA content of HAM and HDM scions, and discovered that both accumulated high levels of GA. However, the GA levels of HAM were markedly higher than those of HM. The large amount of GAs accumulated in HAM may be one of the reasons for the rapid growth of scion.

Further, CTKs promote cell division and enhance plant resistance, which is essential for plant growth. HKs and HPs serve as CTK receptors and respond to stress [53]. In this study, CTK signaling was highly active in HAM vs. HM, and HAM vs. HDM. Specifically, AHP genes were highly expressed. Therefore, the activation of CTK signaling may promote the rapid growth of seedlings. ABA, as a plant growth inhibitor, can regulate the balance of plant moisture by controlling the stomatal closure of the leaf [54]. ABA and auxin regulate stomata through antagonistic action [55]. Some transcription factors in the ABA signaling bind to auxin response genes, thus demonstrating the interaction between auxin and ABA signaling [56]. In ABA signaling, the three crucial elements involved in double-negative transduction are PYL, PP2C and SnRKs. PYR/PYL binds with PP2C to diminish SnRK2 inhibition, hence controlling downstream variables [57]. In our study, multiple ABA response genes, such as PP2Cs, were upregulated in HAM, indicating that PP2C inhibited SnRK2 kinase (a key positive regulator of ABA signaling), inhibited a series of downstream response factors, delayed plant senescence, regulated stomatal opening, enhanced photosynthetic capacity and promoted plant growth. Interstocks mainly cause changes in upstream factors of ABA signaling. Slow-growing rootstocks have been reported to shown higher ABA concentrations than vigorous rootstocks [28]. In this study, the ABA content was the lowest during the vigorous growth period, consistent with the reports of raspberries [58] and peaches [59]. ABA modulates plant growth and fruit ripening by interfering with ethylene- and auxin-related genes [60]. An excess of ABA may delay plant growth rate and cause the plant to enter aging [61]. PP2Cs interact with genes involved in IAA and GA signaling [62]. Therefore, interstocks regulate plant growth by inducing the interaction between IAA, GA and ABA, by mediating PP2Cs expression.

Ethylene produces multiple effects on plant development [63]. In this study, ERF1B was downregulated by interstocks. The ETH-mediated stomatal response is dependent on ABA accumulation, because ABA and ethylene have synergistic effects [64]. High ethylene levels can hinder plant growth [65]. In our study, ERF1B was associated with IAA and PIF4, suggesting crosstalk between ethylene and other plant hormones.

Collectively, our findings showed that interstocks enhanced photosynthesis and improved endogenous hormone metabolism. This was mirrored in the dramatically enhanced transcript levels of a wide range of important genes, resulting in improved light capture to promote photosynthetic efficiency, which may boost nutrient accumulation and transportation, ultimately regulating plant growth.

## 4. Materials and Methods

### 4.1. Plant Materials and Experimental Design

Seeds from the ‘Hongju’ (H) tangerine rootstock were sown in February 2018. Seven months later, when the seedlings were ca. 40 cm in height and the stem was ca. 5 mm thick, a single budwood from mature trees of either ‘Mingrijian’ (M) tangor or ‘Aiyuan 38’ (A) or ‘Daya’ (D) tangerines was grafted onto the ‘Hongju’ seedlings at the height of ca. 10 cm above the ground, forming three groups of rootstock/scion combinations (H/M, H/A and H/D). Then, nineteen months later, in April 2020, a single budwood from mature trees of ‘Mingrijian’ tangor was grafter onto the scion of the H/A and H/D plants, to form interstocked combinations (H/A/M and H/D/M). The schematic diagram of grafting was shown in Figure 1A. The grafted seedlings of ‘Mingrijian’ citrus, including HAM (H/A/M), HDM (H/D/M) and HM (H/M), were grown in plastic pots (28 cm in diameter and 30 cm in depth) for cultivation (one plant per pot). The cultivation substrate was a mixture of nutrient soil and orchard soil at 1:1 (volume ratio) proportions. The soil contained 19.6 g·kg^−1^ organic matter, 109.2 mg·kg^−1^ alkali-hydrolyzed nitrogen, 52.5 mg·kg^−1^ available phosphorus and 64.4 mg·kg^−1^ available potassium. The soil pH was 6.8. None of the grafted plants were infested with pests or infected with diseases, and the plants were characterized by the uniformity of their vigor, soil, and water management in the field. Grafted ‘Mingrijian’ citrus plants were subjected to conventional management outdoors in the agriculture research and development base of Sichuan Agricultural University (30°33′46″ N, 103°39′36″ E). Sixty grafted seedlings from each combination with the same growth potential were selected as experimental materials. Twenty randomly selected seedlings were pooled into one biological replicate, and each combination was analyzed using three biological replicates. Samples of each grafting combination were collected every 30 days from June 2020.

### 4.2. Analysis of Morphological Indicators and Measurement of Physiological Indexes

Growth measurements were performed on new shoots and grafted plants using 30 seedlings per combination (three repetitions per combination; ten seedlings per replicate). The stem diameter of the grafted seedlings (5 cm above the grafting line) was measured using Vernier calipers, and the length of new shoots from base to top and the height of the grafted seedlings from the soil level were measured using a tape measure.

For the determination of chlorophyll, soluble sugar, starch and endogenous hormones, three pools of leaves were collected from 30 grafted seedlings of each grafted combination, at every phase of growth. The leaves used for these measurements were maintained under consistent growth conditions and at similar growth positions. To measure chlorophyll content, expressed as mg·g^−1^, chlorophyll was extracted with 80% acetone and absorbance was measured at 470, 645 and 663 nm [66]. A quantity of 1.0 g of each sample was utilized for extracting the content of soluble sugar. The sample was cut into pieces and placed in 10 mL of water at a temperature of 100 °C. Subsequently, it was subjected to two further extractions with water, at the same volume and temperature. The content of soluble sugar was determined using the NanoDrop 2000C (Thermo Fisher Scientific, Waltham, MA, USA) with the anthrone colorimetric method, at 630 nm [67]. The remaining sample was used for determining the starch content. The tissue residue was digested with perchloric acid at 100 °C in 20 mL of water for 30 min, to convert starch to glucose, which was extracted once again. The anthrone method was used for determining the glucose content [67].

The samples for phytohormones were stored at −80 °C. An enzyme-linked immunosorbent assay (ELISA, Shanghai, China) was used to determine ABA, IAA, GA and CTK, as per previous studies [68]. Fresh leaves of grafted seedlings were collected, weighed as 1.0 g, frozen in liquid nitrogen, and homogenized in a sample extraction buffer. The resulting extract was purified by passing it through C_18_-Sep-Pak cartridges. ELISA reactions were performed using 96-well microtitration plates, wherein each well was coated with 50 μL of sample and 50 μL of antigens against the hormones. The plate was incubated for 30 min at 37 °C. After washing with a buffer, 100 mL of color development solution was added to each well, and the reaction was stopped with the addition of 50 mL of H_2_SO_4_ per well. The microplate reader (BioTek Inc., Winooski, VT, USA) was used to determine the absorbance at a wavelength of 490 nm. All concentrations were measured on the basis of the leaves’ fresh weight (FW).

The net photosynthesis rates (Pn) of the fully expanded fourth leaf, from the top to the base of the new shoots, was determined using the Li-6400 Portable Photosynthesis Analysis System (Li-Cor Inc., Lincoln, NE, USA), from 08:30 to 11:00 a.m.

### 4.3. Anatomical Observation

Three fully mature and healthy leaves with functioning stomata were carefully obtained from the upper-middle section of each grafted seedling. These samples were transported to the laboratory immediately in ice packs, to ensure their freshness. After collecting the stomata through imprinting, images were captured using the Olympus fluorescence microscopy system (BX53 + andorDU888). Randomly selecting three images, we used ImageJ [69] to measure the stomata’s density, length, width and aperture size. Additionally, healthy and fully expanded leaves from 10 individual seedlings of each grafted combination were collected and cut into small pieces (0.5 cm × 0.5 cm), and placed in FAA solution (70% alcohol:glacial acetic acid:formaldehyde = 18:1:1) for 48 h. We prepared transverse sections of the leaves (8 μm thick) using conventional paraffin sectioning [70], stained them with safranin and fast green, and sealed them with optical resin. We then observed and photographed the sections using the Olympus BX53 microscope (Olympus, Tokyo, Japan), measuring various tissue thicknesses including leaf, upper epidermis, palisade tissue, spongy tissue and lower epidermis using ImageJ, and calculating their average values.

### 4.4. Total RNA Extraction and Sequencing

Total RNA of the grafted seedling leaves from HAM, HDM and HM were collected using the RNAiso Plus kit (TaKaRa, Dalian, China). To enact the extraction of total RNA, a quantity equivalent to approximately 1.0 g of fresh leaves from each combination underwent the process of being frozen, ground into a fine powder in liquid nitrogen and homogenized by means of RNAiso Plus solution. In each combination, three replicates were used for RNA sequencing, with the leaves from five potted plants mixed together as a biological replicate. RNA integrity was assessed using the RNA Nano 6000 Assay Kit on the Bioanalyzer 2100 system (Agilent Technologies, Santa Clara, CA, USA), followed by electrophoresis on 1% agarose gel. Library construction and sequencing were performed using the Illumina Hiseq™ 4000 platform (Novogene Biotech Co., Ltd., Beijing, China).

### 4.5. Data Quality Control, Differentially Expressed Gene Analysis and Enrichment Analysis

Raw data (raw reads) from each replication, provided in fastq format, were processed using in-house Perl scripts (Novogene Technologies Co., Ltd., Beijing, China) to obtain clean data (clean reads), by removing low-quality reads and reads containing adapters, and N bases from raw data. The clean data were further analyzed to calculate Q20, Q30 and GC content. Consequently, downstream analyses were performed based on high-quality clean data. The reference genome was indexed using HISAT2 v2.0.5, and paired-end clean reads were matched to the Citrus Clementine genome. Subsequently, the FeatureCounts (1.5.0-p3) software was used to calculate the reads mapped to each gene [71]. Based on the length of the gene and the number of reads belonging to that gene, the FPKM (expected number of fragments per kilobase of transcript sequence per million base pairs sequenced) values were determined for each gene. The DESeq2 software (1.20.0) was used to undertake differential expression analysis between the two comparable groups [72]. The *p*-values were corrected using Benjamini and Hochberg’s method [73]. The *p*-values and |log_2_Foldchange| were utilized as significant differential expression thresholds. For detecting significant differences in gene expression, the *p*-value < 0.05 and |log_2_FoldChange| ≥ 1 were used. Statistical enrichment of DEGs in the KEGG pathway was analyzed using the clusterProfiler (3.4.4) software, whereas log-normalized transcriptome data (Log_2_(FPKM + 1)) were used for heat map analysis. Finally, heat maps of DEGs were created using TBtools [74].

### 4.6. Quantitative Real-Time PCR (qRT-PCR) Validation

RNAprep Pure (Tiangen, Beijing, China) was used to extract total RNA from the scion leaf tissue of HAM, HDM and HM. The RNA reverse-transcription kit (Toyobo, Shanghai, China) was used to form the first-strand cDNA. Primer 5 was used to create unique primers for 12 randomly selected genes, and qRT-PCR analysis was performed to validate the transcriptome results (Table S11). These primers were synthesized by Tsingke Biotech (Beijing, China). To confirm the differential gene expression patterns, qRT-PCR was carried out using Bio-Rad CFX Manager (Bio-Rad, Shanghai, China) and SYBR Premix Ex Taq II (novoprotein, Shanghai, China), with all samples having undergone three biological and technical replications. The 2^−∆∆CT^ method [75] was used to calculate gene expression levels after normalizing them against the geometric mean of the citrus reference gene, *Actin* (GenBank: XM_006429010.2).

### 4.7. Statistical Analysis

A one-way ANOVA was used to evaluate the data for the physiological indexes, followed by the Duncan’s multiple comparisons test. When *p*-value < 0.05 of differences among the grafted combinations were judged as significant. All data were analyzed utilizing the program SPSS 18.0 (SPSS Inc., Chicago, IL, USA) and OriginPro 8.5 (OriginLab Corp., Northampton, MA, USA). The data of three independent biological tests were reported as the means ± standard deviation (SD).

## 5. Conclusions

Our physiological and transcriptome investigations demonstrated that interstocks boosted the development of ‘Mingrijian’ seedlings through diverse pathways. In particular, interstocks enhanced plant growth, biomass accumulation, chlorophyll content and photosynthetic ability. In addition, based on transcriptomic analysis, a majority of the key DEGs related to photosynthesis, photosynthesis–antenna proteins and hormone signaling were expressed or upregulated in the interstocked plants. However, fewer DEGs were detected between HDM and HM, presumably because of the more similar genetic background of ‘Daya’ and ‘Mingrijian’. Additionally, interstocks enhanced the growth of ‘Mingrijian’ seedlings, primarily by regulating photosynthesis and hormone metabolism. Therefore, we determined the expression pattern of key phytohormone signaling pathways and demonstrated the crosstalk between plant hormones in the presence of an interstock. Our results revealed important details on the molecular processes through which interstocks promoted seedling development.

## Data Availability

Transcriptome data are available at the National Center for Biotechnology Information (NCBI) database.

## Supplementary Materials

The following supporting information can be downloaded at: https://www.mdpi.com/article/10.3390/ijms24076533/s1.
