# Supplementary material for: Comparative Transcriptomic and Physiological Analyses Reveal Key Factors for Interstocks to Improve Grafted Seedling Growth in Tangor
Source: Int J Mol Sci. 2023 Mar 31;24(7):6533. doi: 10.3390/ijms24076533 (PMC10095262; doi:10.3390/ijms24076533)
Supplement: Supplementary file 1 [file ijms-24-06533-s001.zip › Supplementary figures.pdf]

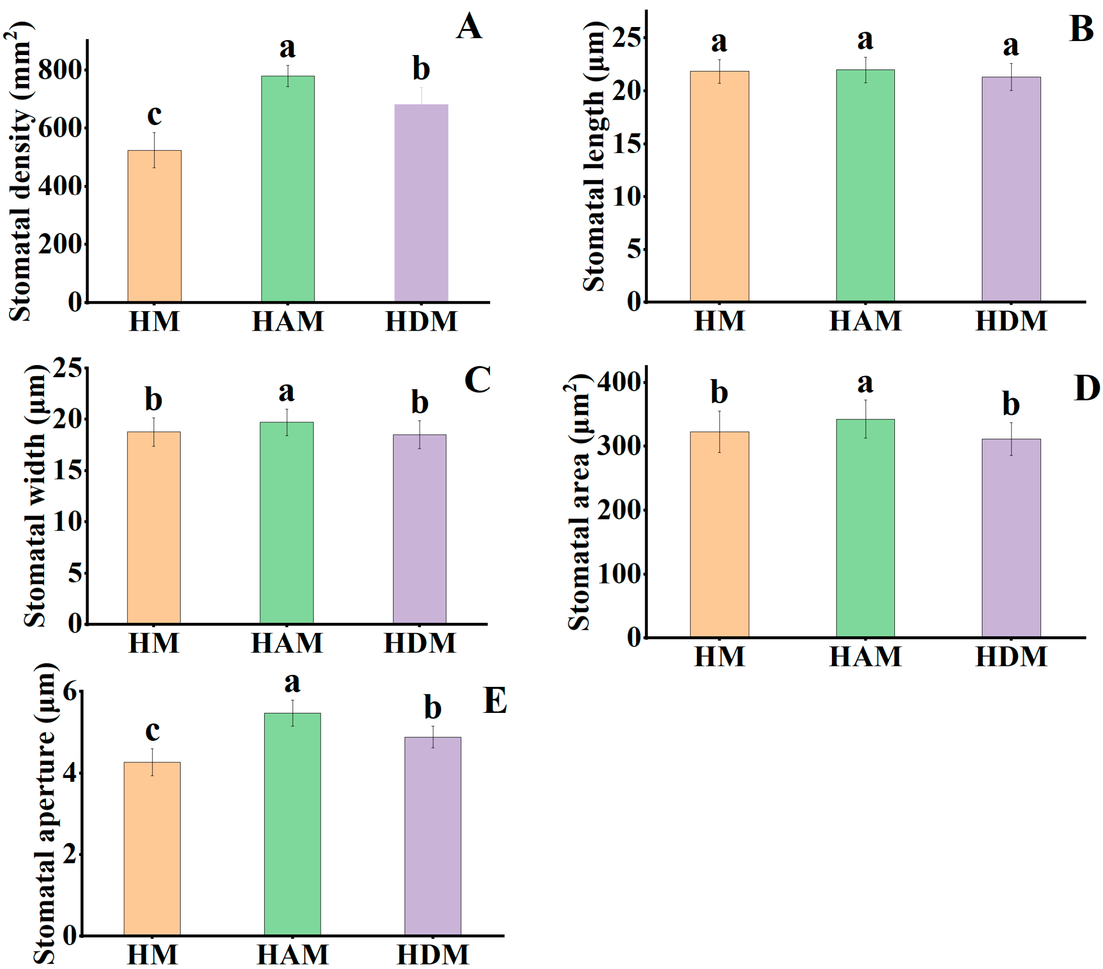

Figure S1. Properties of stomata from plants after 210 days of exposure to different interstocks treatments. (A) Density, (B) length, (C) width, (D) area, and (E) aperture size.

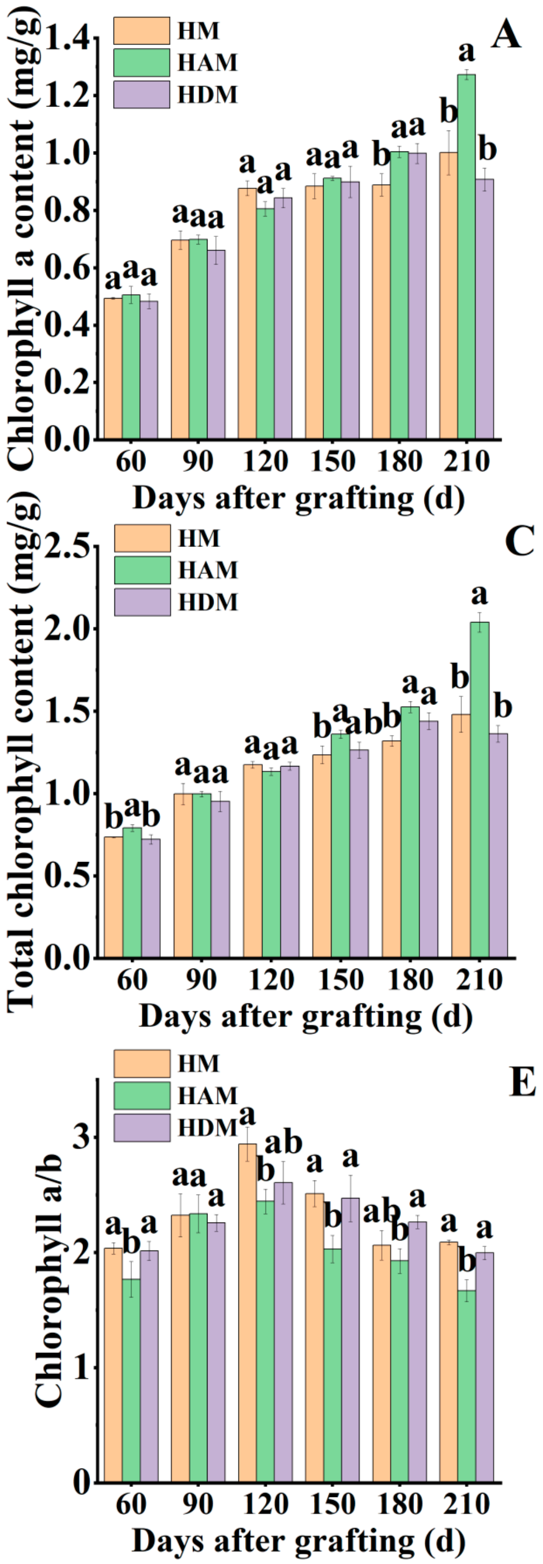

Figure S2. Effect of different interstocks on photosynthetic pigment content of grafted citrus leaves, including (A) chlorophyll a, (B) chlorophyll b, (C) total chlorophyll, (D) carotenoid content, and (E) chlorophyll a/b.

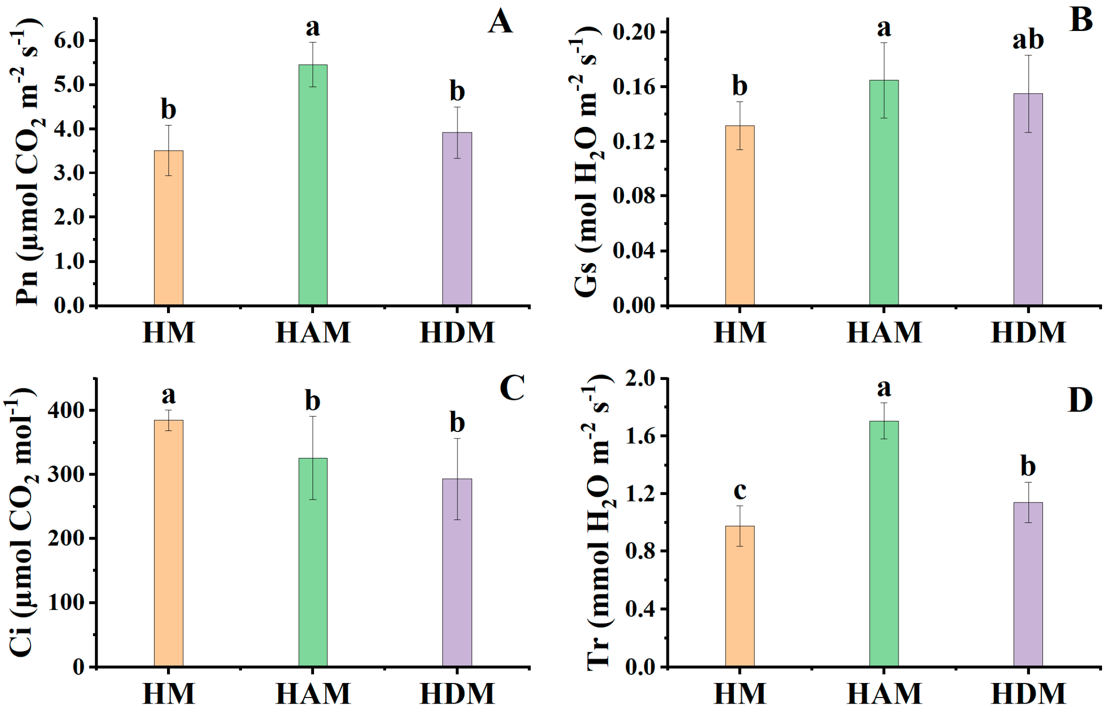

Figure S3. Grafting 'Asumi' seedlings photosynthetic characteristics under different interstocks treatments. (A) Pn, (B) Gs, (C) Ci, (D) Tr.

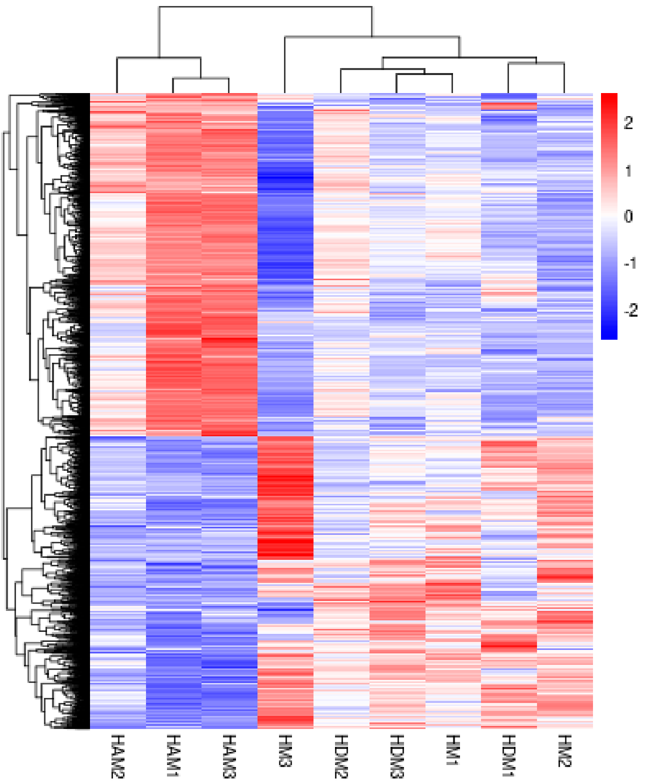

Figure S4. Cluster heat map of differentially expressed genes (DEGs) between different samples. Each column in the graph represents a sample, and each row represents a gene. The color in the graph indicates the amount of gene expression in the sample (log2(FPKM+1)). Red color indicates that the gene is highly expressed in the sample, and green color indicates that the gene is lowly expressed.

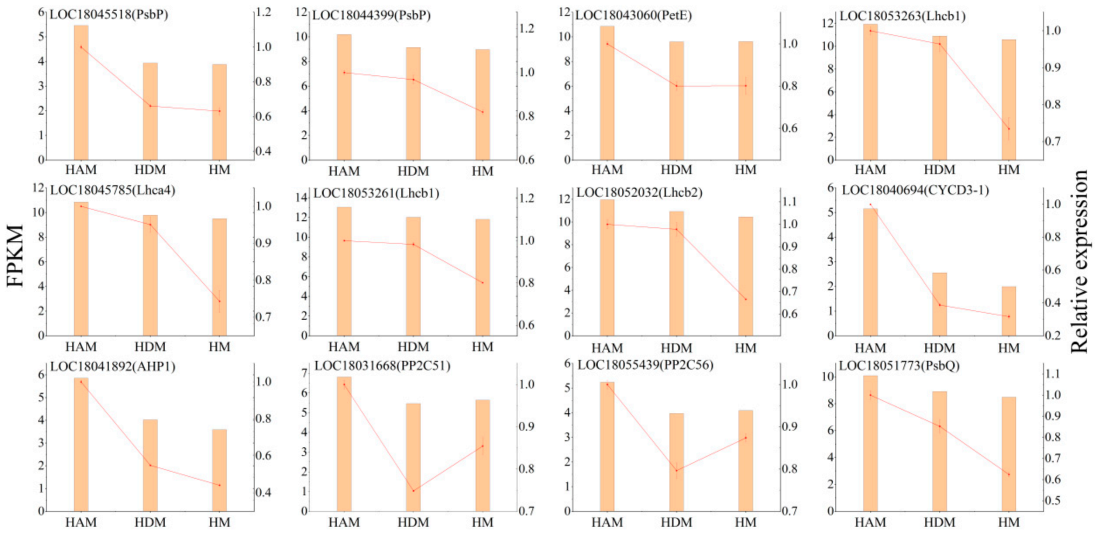

Figure S5. Quantitative RT-PCR analysis was performed on selected DEGS based on the results of RNA sequencing analysis. The left y-axis shows the expression data from RNA-seq (red histogram) analysis. The right y-axis indicates the relative gene expression levels of the corresponding qRT-PCR analysis (red line). X-axis indicates samples from different grafting combinations.

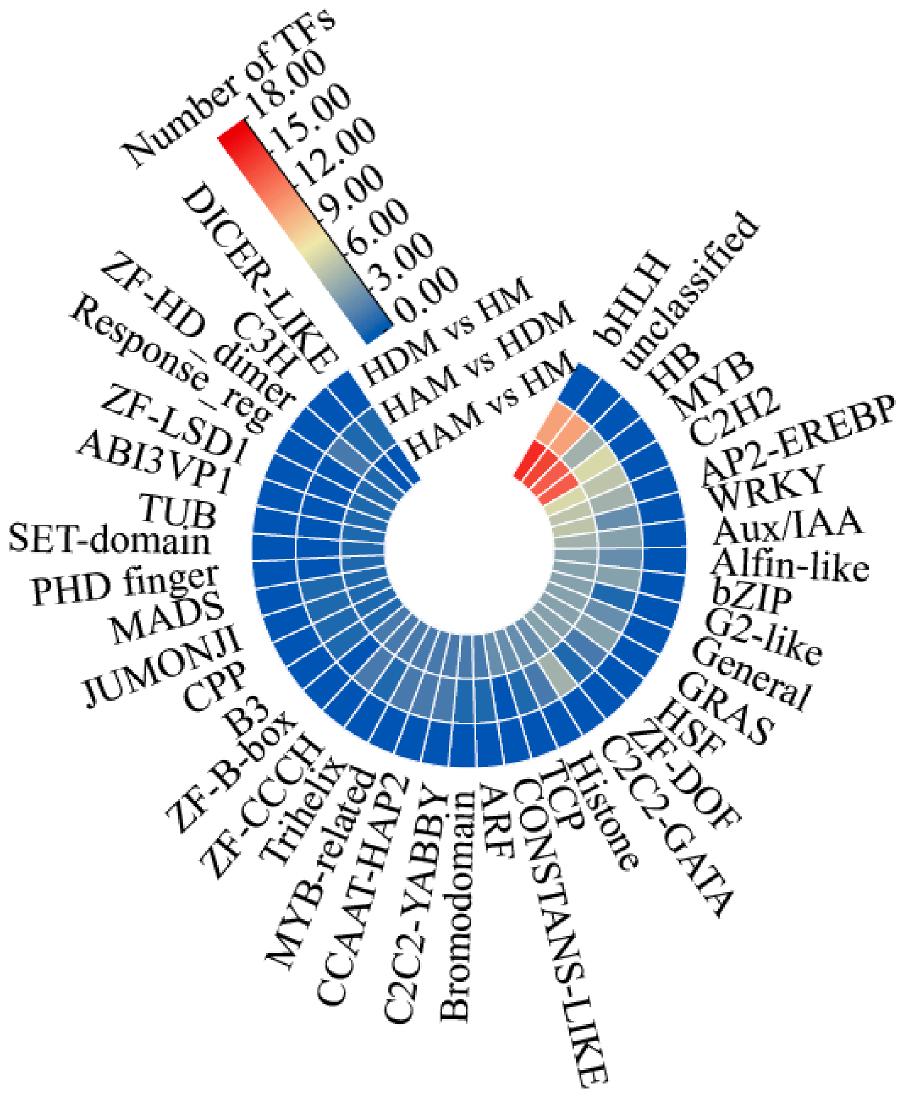

Figure S6. Heatmap of TF families in three comparisons.

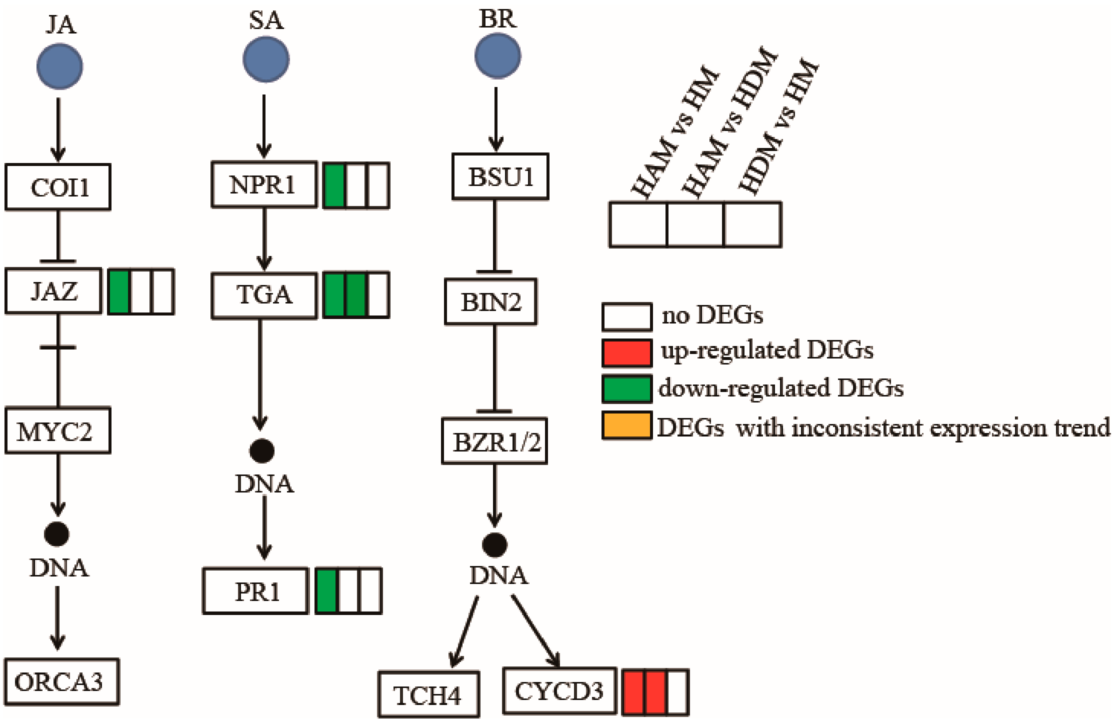

Figure S7. Interstocks induced changes in the expression profiles of hormone signaling pathway genes in three comparisons. The rectangle behind the gene, which was tagged with red, green, white, and yellow color, represent the upregulated DEGs, downregulated DEGs, unchanged DEGs, and the DEGs with inconsistent expression trends, respectively.
